# Isolation and characterization of a new simian rotavirus, YK-1

**DOI:** 10.1186/1743-422X-3-40

**Published:** 2006-05-31

**Authors:** Larry E Westerman, Baoming Jiang, Harold M McClure, Lauren J Snipes-Magaldi, Dixie D Griffin, Gary Shin, Jon R Gentsch, Roger I Glass

**Affiliations:** 1 Viral Gastroenteritis Team, Respiratory and Enteric Viruses Branch, Centers for Disease Control and Prevention, Atlanta, Georgia, USA; 2 Yerkes National Primate Research Center, Emory University, Atlanta, Georgia, USA; 3 Department of Ecology and Evolutionary Biology, University of California at Los Angeles. Los Angeles, California, USA

## Abstract

**Background:**

To effectively analyze the requirements for protection to rotavirus infection, a reliable animal model that reasonably mimics infection and disease in humans is needed. A requirement for an effective animal model is the availability of appropriate rotavirus stocks for challenge.

**Results:**

A new simian rotavirus, designated YK-1, was isolated from a 2-year-old immunodeficient pigtailed macaque with chronic diarrhea. YK-1 was distinguishable by electropherotype from the other simian rotavirus strains, SA11 and RRV. One variant of YK-1, clone 311, which was isolated after adaptation and plaque purification in cell cultures, displayed an unusual RNA electropherotype with an abnormally migrating gene 11 segment. Sequence analysis demonstrated a genetic rearrangement that involved a partial duplication of the gene 11 ORF encoding NSP5. YK-1 was identified as a Group A rotavirus belonging to subgroup 1. To further characterize the YK-1 strain, the genes encoding VP4, VP7, and NSP4 were sequenced. Analysis of VP4 and VP7 gene fragments suggests that this strain is a G3P[3] rotavirus and is closely related to the simian rotavirus strain RRV. Serotype analysis also identified YK-1 as a G3 rotavirus. The NSP4 genotype of YK-1 is C, the same genotype as RRV.

**Conclusion:**

This newly isolated rotavirus, YK-1, is being used to establish a nonhuman primate model for studying the infectivity, immunity, and pathogenesis of rotavirus and for evaluating candidate rotavirus vaccines.

## Background

Rotaviruses have a wide host range and can be recovered from many animal species [1]. The ability to isolate and maintain rotaviruses and to use them in animal model systems has contributed to studies of the mechanisms of pathogenesis and immunity and to the development of vaccines. Several rotavirus isolates and animal model systems have been successfully developed, including various murine rotavirus strains in infant and adult mice [2-4], C11 and Ala strains in rabbits [5], and human rotaviruses with piglets [6].

Two simian rotavirus strains, SA11 and RRV, have been well characterized and are currently the most widely used reference strains in laboratories throughout the world [7-9]. The sequences of all 11 genomic segments of SA11 are available. In limited studies, rotavirus infection and disease have been induced in nonhuman primates inoculated with SA11 [10-13]. Also, some human rotavirus vaccines are based on the RRV strain or reassortants of RRV with human strains [14]. The use of simian strains in human vaccines was based on a Jennerianapproach prompted by studies indicating that animal and human rotaviruses share a common group antigen and that experimental animals immunized with human strains of rotavirus had a significantly lower risk of disease and infectivity when subsequently challenged with animal rotaviruses [15].

Although the two well-characterized simian rotavirus strains are readily available for use as a challenge virus, they have not been used consistently in nonhuman primate models of rotavirus infection because of their numerous passages in cell culture, which is a common method for viral attenuation. We wanted to isolate and characterize a new simian virus that is with low passage number in cell culture to be used as a challenge virus in nonhuman primates. The isolate strain, designated YK-1 and its variant clone 311, is being used to establish a nonhuman primate model for studying the infectivity, immunity, and pathogenesis of rotavirus and for evaluating candidate rotavirus vaccines [16,17].

## Results

### Rotavirus isolation and characterization

**Figure 1:**
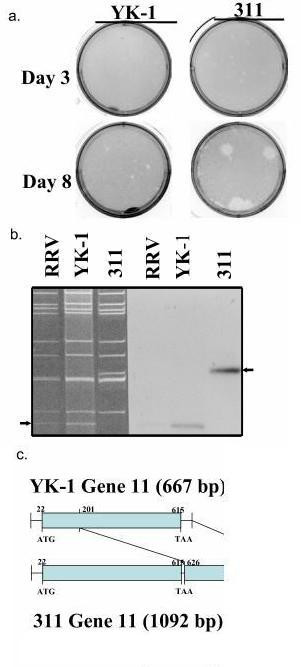
Comparison between the YK-1 strain and its variant 311 by (a) plaques size, (b) Northern blot analysis for rotavirus RNA segment 11, and (c) schematic diagram of the sequences of gene segment 11. Arrows indicate segment 11 of RRV and YK-1, or rearranged segment 11 of variant 311.

High titers of rotavirus antigen measured by an immunoassay were consistently detected in the stools of an immunodeficient pigtailed macaque, PFm-1, which was infected naturally with rotavirus and developed severe, chronic diarrhea. Rotavirus-like virus particles were detected by electron microscopy in stool extracts from this macaque (data not shown). Polyacrylamide gel electrophoresis of the viral RNA segments extracted from a stool specimen from PFm-1 revealed an electrophoretic pattern consistent with other rotaviruses, except for a lower intensity of segment 11 and an additional segment migrating slightly slower than segments 7, 8, and 9 (data not shown). This RNA electropherotype suggested that the stool extract was a mixture of subpopulations of rotavirus, as reported for other human rotavirus isolates with genome rearrangements [18,19]. The rotaviruses from PFm-1's stool extract were adapted to grow in MA-104 and plaque purified, which revealed two distinct viruses, named YK-1 and clone 311, that were distinguishable by plaque size and electropherotype (Figure 1a and 1b) [20,21]. YK-1 produced smaller plaques and had an electropherotype typical of group A rotaviruses and that was very similar to but distinct from RRV. Variant 311 produced larger plaques and had an RNA electropherotype identical to that for YK-1, except it had an additional segment that was migrating slightly slower than segments 7, 8, and 9 and did not have a typical migrating segment 11. Both YK-1 and 311 showed a cytopathic effect typical of rotavirus grown in MA-104 cells and readily grew to titers over 10_8 _ffu per ml.

### Variant 311 has a rearranged segment 11

The nucleotide sequence of segment 11 from YK-1 was first determined as a reference. It consisted of 667 nt with a 594-bp ORF flanked by 5' and 3' UTRs of 21 and 52 nt, respectively. Segment 11 of YK-1 had 99% similarity to segment 11 of RRV. In the variant 311, segment 11 migrated slower than that of YK-1 and RRV, as determined by Northern blot analysis with a probe specific for segment 11 (Figure 1b). Sequence analysis of segment 11 from variant 311 identified a rearrangement consisting of a partial duplication of segment 11 from YK-1. The rearrangement occurred at nt 626 in the 3' UTR with the duplication of the ORF starting at nt 201 and included the entire 3' UTR (Figure 1c). The sequence of variant 311's segment 11 has 100% identity to YK-1's segment 11 both in the ORF and in the partial duplication.

### YK-1 group, subgroup, and serotype analyses

The VP6 protein of rotavirus confers group specificity that is divided into seven groups (A to G). The commercial immunoassay Rotaclone utilizes a monoclonal antibody directed against the group A VP6 antigen and identified YK-1 as a group A rotavirus [22]. Group A rotavirus strains have been separated into four subgroups, and YK-1 is designated subgroup 1 as determined by reactivity with a subgroup 1 MAb 255/60 but not with subgroup 2 MAb 631/9.

**Figure 2:**
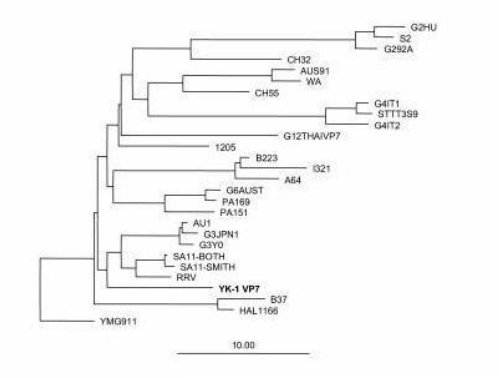
Phylogentic tree based on amino acid sequences of the VP7-encoding genes for YK-1 and other established rotavirus strains.

**Figure 3:**
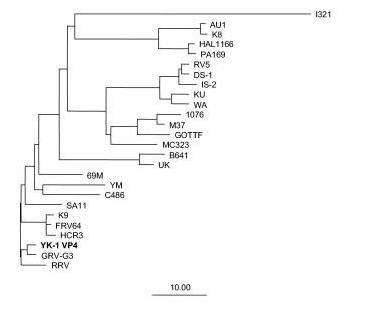
Phylogentic tree based on amino acid sequences of the VP4-encoding genes for YK-1 and other established rotavirus strains.

To predict the G and P serotype specificities of the YK-1 strain, the sequences of the genes encoding both VP7 and VP4 were determined and compared with those of representatives of established G and P serotypes. The deduced VP7 amino acid sequence of YK-1 was closely related to other simian G3 rotavirus strains: 89% amino acid identity with RRV and 88% amino acid identity with SA11. A phylogenetic tree was constructed that included known VP7 amino acid sequences of G3 and other common G serotypes (Figure 2). The YK-1 strain clustered with other strains of G3 serotype, including RRV, SA11, AU1, and YO. YK-1 was also identified as a G3 serotype by reactivity with MAbs YO-1E2 (G3) and G3-159 (G3) in an immunoassay utilizing MAbs reactive to VP7-specific protein. The predicted VP4 amino acid sequence of YK-1 closely resembled that of GRV, a newly identified P[3]G3 caprine strain, and that of RRV P[3]G3 (Figure 3) [23].

### NSP4 sequence analysis

**Figure 4:**
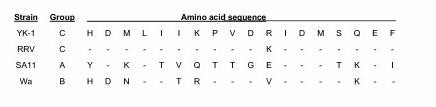
Comparision of the NSP4 deduced amino acid sequences at the variable portion in the VP4- binding domain (aa 131–148) from representative groups of rotavirus strains.

The NSP4 gene segment of YK-1 was sequenced and its deduced gene product was compared with those of other known rotavirus strains. The structure of the NSP4 gene of YK-1 was similar to those of other rotavirus strains that were sequenced previously. The gene consists of a 528-bp ORF that encodes a protein with a predicted size of 175 amino acids with two conserved potential N-linked glycosylation sites. The deduced amino acid sequence of YK-1's NSP4 was 98% similar to that of the simian RRV strain. At least four genetic NSP4 groups are known, and the NSP4 of YK-1 can be classified as Group C by comparison of the amino acid sequence (aa 131–148) of the variable portion in the VP4 binding domain of various groups of NSP4 (Figure 4) [24,25].

## Discussion

A rotavirus infection model using nonhuman primates offers a highly relevant system to investigate the mechanisms of disease and immunity to rotavirus and to determine vaccine effectiveness [16,17,26]. Since nonhuman primates are the animals most closely related to humans, this model may be the best predictor of infection and immunity in humans. In order to perform such studies, it is necessary to have a rotavirus strain that will consistently infect nonhuman primates after oral challenge. We describe rotavirus isolates that were obtained from a naturally infected pigtailed macaque housed in a major primate research center. This monkey was immunosuppressed and had severe chronic diarrhea possibly due to the rotavirus infection. Two isolates were obtained from a stool of this monkey and one designated YK-1 had an electrophoerotype typical of most group A rotaviruses and the other, designated 311, was identical to YK-1 except for a rearrangement in gene segment 11 that encodes for the NSP5 protein.

The YK-1 and its variant clone 311 were fully adapted to grow in cell culture, and both strains could produce plaques on MA-104 cells, although the plaques from the 311 variant were larger than those from the YK-1 strain. The significance of the difference in plaques size is not known. Both YK-1 and 311 were identified as a group A rotavirus, subgroup 1, genotype P[3] and serotype G3. It is of interest that these characterizations of YK-1's G and P types are very similar to another simian rotavirus strain, RRV. The nucleotide sequence of the NSP4 gene from YK-1 was also determined because of the discovery of the NSP4 gene product as a viral enterotoxin and its implication in the virulence of rotavirus [27]. YK-1 was determined to have a group C NSP4 gene, which again was similar to that of the RRV strain.

Group A rotaviruses with atypical RNA profiles due to genomic rearrangements have been repeatedly detected in stools of chronically infected immunodeficient children [18,28]. These types of rearrangements have also been detected in rotavirus isolates from apparently immunocompetent calves and rabbits [29-31]. We have also isolated an YK-1 variant, 311, with a rearrangement in gene segment 11. With these rotaviruses, the rearrangement results from a partial duplication of the gene with a normal 5' UTR followed by a normal ORF and a duplication starting at various positions after the stop codon and extending to the 3' end and leading to a long 3' UTR. Thus, the rearranged gene expresses a normal protein product. Although the function of this rearrangement is unknown, it has been proposed to play a part in the evolution of rotaviruses and to contribute to their diversity [32]. It has also been suggested that rearranged segments containing a partial duplication might be more efficient templates for double stranded RNA synthesis than are their wild-type counterparts and thus may be preferentially selected during viral replication [31].

## Conclusion

Development of a more suitable animal model of rotavirus infection requires the identification of an appropriate challenge strain. The ideal challenge virus should be isolated from the same species as that employed in the model system because, in some systems, heterologous rotaviruses tend to undergo abortive replication. We have isolated a new rotavirus strain, designated YK-1, from fecal specimens of a 2-year-old pigtailed macaque with severe chronic diarrhea. The YK-1 strain had been used to develop a nonhuman primate model to enhance our understanding of the mechanisms of immunity to rotavirus infection [16,17]. This report describes the characterization of this new strain and a variant of this strain and compares the properties of this strain to those of the other simian rotavirus strains, SA11 and RRV.

## Materials and methods

### Rotavirus isolation

The YK-1 strain of simian rotavirus was isolated from the diarrheal stool of a 2-year-old pigtailed macaque (*Macaca nemestrina*) housed at the Yerkes National Primate Research Center, Emory University (Atlanta, GA). This immunodeficent macaque, PFm-1, had chronic diarrhea associated with high titers of fecal rotavirus antigen detected by Rotaclone immunoassay (Meridian Diagnostics, Cincinnati, OH). The virus was isolated by previously described methods with modifications [20,21]. An extract from an antigen positive stool was prepared as a 20% (wt/vol) suspension in phosphate buffered saline (PBS, pH 7.4) and centrifuged twice at 8500 *g *for 10 min for clarification. The supernatant was extracted with 1,1,2-trichlorotrifluoroethane (Sigma, St. Louis, MO), and centrifuged at 4000 × g for 5 minutes. The extract was treated with tryspin (15 _g/ml) for 45 min at 37°C and inoculated onto a confluent monolayer of MA104 cells (African green monkey kidney cells) for 1 h. After being washed, the monolayer was maintained in serum-free minimal essential medium (MEM) (Gibco, Grand Island, NY) supplemented with 2 _g/ml tryspin and 50 _g/ml neomycin for 3 days. A viral lysate, obtained by freeze-thawing three times and clarification at 8500 *g *for 30 min, was inoculated into MA104 cells and plaque purified three times. Two distinct plaques distinguished on size were obtained and further passed in MA104 cells.

### Plaque assay

Virus stocks were activated with 15 _g/ml tryspin in MEM for 45 min at 37°C. The activated virus was 10-fold serially diluted in MEM and 500 _l/well was inoculated onto 6-well tissue culture plates (Corning, Corning, NY) with a confluent monolayer of MA104. After a 1 h incubation at 37°C, the inoculum was aspirated and 4 ml of a 3.5% agarose (Seakem, Biowhittaker, Rockland, ME) in MEM was overlayed on the monolayer. The agar was allowed to solidify at room temperature (RT), after which the plates were incubated at 37°C. Plaques were visualized by adding 1 ml of MEM with 2% neutral red and 0.3% agarose 6 h prior to reading.

### Purification of virus RNA

Rotavirus RNA was extracted from stools and infected cell cultures by a modification of a previously described method [33]. In brief, a 10% stool extract or 30% cell-culture suspension was prepared with Tris-buffered saline supplemented with 1% sodium dodecyl sulfate (SDS), vortexed, and incubated at RT for 10 min. Equal volumes of virus and 1,1,2-trichlorotrifluoroethane were mixed for 1 minute, and centrifuged for at 8000 *g *for 10 min. The supernatant was added to 2 volumes of 6 M guanidine isothyiocyanate and incubated at 56°C for 10 min. Silica beads were added to each sample, vortexed, and incubated at RT for 10 min. The beads were washed once with a 2:1 solution of 6 M guanidine isothyiocyanate with 50 mM Tris-HCl (pH 7.5) and then three times with 70% ethanol. After the final wash, the beads were air dried, incubated with H_2_O for 10 minutes at 65°C, and centrifuged for 2 min at 10,000 *g*. The extract was saved and stored at -70°C until use.

### Electropherotyping

Rotavirus double-stranded genomic RNA extracted from fecal samples and cell-culture lysates were analyzed by SDS-polyacrylamide gel electrophoresis as described previously [34].

### Northern blot for segment 11

The procedures employed for Northern hybridization and chemiluminescent detection of bound digoxigenin-labeled probe using a commercial reagent (ECL, Amersham, Piccataway, NJ) have been described [35]. Two digoxigenin-labeled probes, ggcttttaaagcgctacagtgatgt and ggtcacaaaacgggagtggggagctcc, were used to identify genomic segment 11 of rotavirus.

### Subgroup and serotype analyses

Subgroup and VP7 serotyping were determined by use of a panel of monoclonal antibodies: 225/60 (Subgroup I), 631/9 (Subgroup II), KU-4 (G1), 5E8 (G1), S2-SG10 (G2), IC10 (G2), YO-1E2 (G3), G3-159 (G3), and ST-2G7 (G4) [36-38]. In brief, Immulon II plates (Nagle Nunc, Rochester, NY) were coated overnight with serum from a rabbit hyperimmunized with purified RRV rotavirus particles for positive wells and normal rabbit sera for negative wells. After the plate was washed with wash buffer (PBS plus 0.1% Tween 20), cell-culture lysates were added to duplicate positive and negative wells. The plates were incubated for 2 h at RT and washed. Specific monoclonal antibodies were added to wells, and the plates were incubated for 1 h at RT and then washed. Biotinylated goat anti-mouse IgG (Southern Biotechnology, Birmingham, Al) was added, incubated 30 min at RT and followed by washing and the addition of strepavidin-horseradish peroxidase (Southern Biotchnology). Wells were developed by adding tetramethylbenzidine (Sigma) and stopped after 10 min with 1 N HCl. A sample was considered positive if the OD value of the positive coated well was >2 times and 0.100 greater than the negative coated well.

#### PCR amplification and sequence analysis of VP4, VP7, NSP4, and NSP5

The PCR products of the genes coding for VP4, VP7, and NSP4 proteins were amplified with previously described primers, Con2/Con3 for VP4, Beg9/End9 for VP7 and 10Beg16/10End722 for NSP4 [33,39,40]. Full-length PCR product for the YK-1 gene coding the NSP5 protein was amplified with primers derived from the 5' and 3' ends of the NSP5 nucleotide sequence of the SA11 strain. The nucleotide sequence of each gene was determined from gel-purified PCR products as previously described [41]. Phylogentic relatedness of the VP4 and VP7 genes of YK-1 was examined by comparing amino acid sequences between reference rotavirus strains by using the Wisconsin Genetics Computer Group computer program [42].

## Competing interests

The author(s) declare that they have no competing interests.

## Authors' contributions

LEW characterized and maintained the YK-1 virus and drafted the manuscript, BJ helped draft the manuscript, HMM provided samples for virus isolation, LJSM isolated YK-1, GS and DDG sequenced YK-1, JRG provided phylogenic analysis of YK-1, and RIG helped draft and critically review the manuscript.
